# Multifactorial Inspiratory Muscle Training in Patients With Diabetic Polyneuropathy: A Qualitative Study

**DOI:** 10.1155/jdr/6670842

**Published:** 2026-04-17

**Authors:** Suman Sheraz, Arshad Nawaz Malik, Francesco Vincenzo Ferraro, Furqan Ahmed Siddiqi

**Affiliations:** ^1^ Faculty of Rehabilitation and Allied Health Sciences, Riphah International University, Islamabad, Pakistan, riphah.edu.pk; ^2^ Clinical Exercise and Rehabilitation Research Centre, University of Derby, Derby, UK, derby.ac.uk; ^3^ Foundation University College of Physical Therapy, Foundation University Islamabad, Islamabad, Pakistan, fui.edu.pk

**Keywords:** diabetic polyneuropathy, health-related quality of life (HrQoL), patient′s perceptions, Type II diabetes mellitus

## Abstract

The aim of this descriptive qualitative study was to explore the patient′s perspective of multifactorial home‐based inspiratory muscle training (IMT) combined with group‐based Otago Exercise Program (OEP) to bridge the gap of the experiences and perceptions of diabetes polyneuropathy patients regarding home‐based multifactorial IMT intervention. Individual semistructured interviews were conducted with 12 patients using thematic analysis. The patients′ interviews were transcribed verbatim in Urdu, translated into English, and then coded into relevant themes. Three key themes emerged, including perception regarding quality of life, facilitators, and barriers to intervention. The patients reported improvement in their quality of life perceived through improvement in their physical and mental health as well as their functional independence, while improvement in shortness of breath and walking capacity, discomfort, initial difficulty in usage and distraction in the home environment were the key barriers. A multifactorial IMT intervention was perceived to have positive effects on the physical, mental, and emotional health of diabetes patients. These findings highlight the importance of multifactorial IMT interventions for patients with diabetic polyneuropathy and suggest tailoring interventions and physical therapies to address the barriers and facilitators to enhance the likelihood of successful training outcomes.

## 1. Introduction

Patients′ perspectives on diabetes mellitus (DM) highlight the fears of patients concerning fatigue, generalized body pains, troublesome excessive urination, and dread of experiencing complications if they do not adhere to medical treatment [1]. DM, in particular Type II DM, is a chronic metabolic disease characterized by persistent hyperglycemia owing to insulin resistance of the body. Current physical therapy goals for the management of DM are targeted toward the prevention of complications, which occur more often with uncontrolled blood glucose levels [2, 3]. Among the chronic complications of diabetes, diabetic polyneuropathy (DPN) is highly prevalent affecting about 50% of patients with diabetes and it significantly impacts patients′ functional mobility and quality of life. DPN typically presents with peripheral nerve damage, causing symptoms like pain, paresthesia, numbness, and balance impairments—particularly in the distal lower limbs [4]. As reported in NICE guidelines, multiple management strategies are used to control the glycemic index and manage comorbidities and complications such as DPN [5–7].

The American College of Sports Medicine (ACSM) and the Centers for Disease Control and Prevention (CDC) suggested at least 30 min of moderate‐intensity physical activity (PA) on most days of the week for adequate glycemic management [8]. Multiple exercise training protocols, including aerobic [9], resistance [10], high‐intensity interval training [8, 9, 11], Tai Chi [12], and calisthenics [13], are currently being used for a range of effects in diabetic patients. A recent systematic review (2023) suggested that inspiratory muscle training (IMT), defined as a therapeutic technique to improve the strength of respiratory muscle [14], is an effective intervention especially when in addition to other exercise forms specific to the needs of the patients [15]. IMT intervention can be provided through normocapnic hyperpnea, flow‐resistive devices, and pressure threshold devices out of which pressure threshold devices are most commonly used in research and clinical practice.

The IMT has been used with COPD [16], heart failure [17], and stroke [18] and is currently considered for its effects on balance with the elderly [19], stroke [20], heart failure [21], and in DM [22]. IMT is particularly relevant for individuals with DPN, as this complication can affect not only peripheral nerves in the limbs but also respiratory muscles due to autonomic and motor nerve involvement [23]. Reduced respiratory muscle strength may contribute to fatigue, impaired physical function, and lower exercise tolerance in this population. IMT has the potential to improve respiratory efficiency, trunk stability, and postural control, all of which are often compromised in patients with DPN and may contribute to improved functional mobility and health‐related quality of life (HrQoL). However, there is an evident research gap in terms of patients′ perceptions of this training. Blanco et al. reported improvement in symptoms of patients and performance of activities of daily living in patients with interstitial lung diseases [24]. Another study by Domingo et al. reported positive perceptions of a supervised home‐based IMT program in patients post COVID‐19 [25]. The patients′ views with this easy‐to‐perform home‐based intervention will provide a real‐world perspective and will help healthcare practitioners adapt strategies based on the individualized needs and preferences of patients improving the care quality [26].

The aim of the current study is to report patients′ perspectives on a multifactorial IMT‐based exercise program designed to improve balance, diminish fear of falls, and increase functional mobility in patients with DPN [27].

## 2. Methods

### 2.1. Research Design and Methods

The current study utilizes qualitative research methods to explore the perception of patients toward home‐based multifactorial IMT. Semistructured interviews were conducted among patients with DM, using an interview guide having an open‐ended questioning approach. The consolidated criteria for reporting qualitative research (COREQ) were used to ensure detailed and comprehensive reporting [28]. All the experiments were performed in accordance with relevant guidelines. A preprint of this research has previously been published [29].

### 2.2. Sample Size

The data was collected from 12 patients with DM (who underwent multifactorial intervention) until saturation of data. The patients were recruited from December 2022 until March 2023.

### 2.3. Participants

Participants who were > 50 years of age and have participated in 12 weeks of multifactorial IMT were selected through nonprobability purposive sampling. The patients were diagnosed with Type II DM and were having polyneuropathy confirmed by the modified Toronto Clinical Neuropathy Score (mTCNS). They had their Berg Balance Scores ranging from 30 to 50. The patients having uncontrolled diabetes, hypertension, and disease exacerbation in the last 3 months or having musculoskeletal comorbid conditions (low back pain, osteoarthritis, etc.) were not selected for intervention. Those patients who were not able to communicate in Urdu or were not able to complete 12 weeks of intervention were not interviewed.

### 2.4. Ethical Considerations

This study was approved by the ethical committee of Riphah International University, Islamabad, Pakistan (Ref # Riphah/RCRS/REC/13564). Written informed consent was taken from all the patients prior to the data collection.

### 2.5. Multifactorial IMT Intervention

The home‐based multifactorial IMT is composed of 12 weeks of an established protocol of IMT along with the Otago Exercise Program (OEP) and a standardized protocol for patients with diabetes as per the recommendation of ACSM for 12 weeks [22]. IMT was performed twice daily at an initial load of approximately 50% MIP, and it was progressively increased as per the validated published protocol [22]. While IMT was the core component of this study, additional exercise elements—specifically the ACSM‐recommended diabetes exercise guidelines and the OEP—were integrated to address ethical and clinical needs related to diabetes management and balance deficits in this population. The patients performed the intervention at home, but one supervised session was conducted once a week to check progression in the intervention. The record for adherence to the treatment protocol was maintained through a training diary which was monitored weekly and the patients with < 80% adherence rate were considered dropouts.

### 2.6. Data Collection Procedure

The participants were briefed about the study, and a preferred time for an individual semistructured interview was taken from the patients. The interviews were conducted with 12 patients, and data was collected till the point of saturation after which no further new information was being added [30]. The interview‐based meeting was audio recorded after getting permission from the patients. An interview guide was prepared prior to data collection through the contribution of all authors and piloted on two patients before conducting interviews with the patients. The questions uncovered the experience and perception with IMT, the perceived effect of training, and the challenges or factors enhancing exercise performance. All interviews were conducted by one female interviewer (S.S.) having experience in conducting interviews. The interviews lasted for approximately 30 min (range 20–40 min). All interviews were conducted in Pakistan Railway General Hospital following intervention, in the local language (Urdu), and were recorded and transcribed verbatim for the analysis.

### 2.7. Data Availability Statement

The datasets used and analyzed during the current study are available from the corresponding author on reasonable request.

### 2.8. Data Analysis Procedure

The interviews from the patients were transcribed verbatim by one author (S.S.), rechecked for accuracy, and then analyzed following principles of reflexive thematic analysis [31, 32]. The process involved deep immersion in data followed by a rigorous and systematic process to generate themes [31]. All the interviews were conducted by the primary data analyst (S.S.) who not only conducted all the interviews but also supervised the 12‐week intervention. She recorded her opinions, thoughts, and reflections of the patients and their engagement in the intervention through informal observation and was therefore deeply immersed in data over a prolonged period utilizing the opportunity for strong rapport building with the patients. This reflective process enabled S.S. to acknowledge her biases regarding the intervention based on her role and prior knowledge. She drew on these reflections, informing discussion about the data with other members involved in the analysis, which enhanced the rigor of the analysis process.

S.S. thoroughly read and reread the transcribed data to become familiar with the data, identify patterns to gain insight into every patient′s perception and experience with intervention. She then developed an initial list of codes thereby organizing the data and later collating the codes; she developed subthemes and eventually themes from it [33]. Another author (A.N.M.) did a cross‐examination of the data by reverse tracing verbatim quotations back to the transcripts to ensure that the developed themes were grounded in the original data [34]. Ascertaining methodological rigor, themes, and quotations were then reviewed by another author (F.A.S.) to validate their significance and offer an alternative interpretation of the data [35, 36]. This process continued through multiple iterations until an acceptable consensus was reached by the research group. The interpretation and analysis of the data thereby involved the inclusion of multiple perspectives that encompassed the viewpoints of both participants and analysts. The data was translated into English after the analysis and careful selection of quotes from the local language (Urdu). To ensure translation validity when translating complex constructs, the authors consulted with each other and with a professional English translator [37].

## 3. Results

A total of 12 patients with DPN (seven men and five women, of age ranging between 50 and 66 years) were interviewed in the study. For all the patients, the disease was diagnosed for more than 8 years, and all the patients had moderate to severe levels of DPN (mTCNS score ranging from 9 to 12). The demographic and clinical characteristics of patients are mentioned in Table 1.

**Table 1:** Demographic and clinical characteristics of patients.

	**Age (years)**	**Gender (M/F)**	**BMI (kg/m** ^**2**^ **)**	**Duration of diagnosis (years)**	**Treatment (medicines/insulin/both)**	**mTCNS score**
P1	56	M	30.1	12	Insulin	10
P2	56	F	34.9	8	Medicines	12
P3	65	M	28.3	9	Medicines	10
P4	52	F	26.7	8	Both	9
P5	53	F	29.5	11	Medicines	9
P6	66	M	26.8	9	Insulin	12
P7	60	M	31.6	8	Both	12
P8	56	M	22.3	14	Insulin	9
P9	55	M	29.4	16	Insulin	10
P10	63	M	25.1	14	Insulin	11
P11	50	F	32.2	8	Medicines	10
P12	55	F	25.9	12	Medicines	9

Abbreviations: BMI, body mass index; mTCNS, modified Toronto Clinical Neuropathy Scoring System.

Three key themes were identified after data analysis: (a) perception of quality of life, (b) facilitators, and (c) barriers to home‐based multifactorial IMT. The quotations from the data are highlighted below with the description of each theme. A detailed description of themes, subthemes, and codes is given in Table 2.

**Table 2:** Themes, subthemes, and codes related to patients′ perception of multifactorial IMT.

**Theme**	**Subthemes**	**Codes**
Improvement in HrQoL	Experienced better breathing control	• Decreased SOB during activity • Level of dyspnea reduced
	Generalized improvement in physical health	• Improved walking capacity • Decreased level of fatigue • Feeling of relaxation • Better sleep • Functional independence
	Reduced physical discomfort	• Decreased muscle pain and stiffness • Reduced muscle aching
Facilitators	User‐friendly regimen	• Safe and easy to use • Needs minimal supervision • Easy customization of intervention • Easy incorporation in daily routine • Saves travel time • Flexible schedule
	Reduced healthcare cost	• Saves travel cost • Saves per session cost • Reduced financial burden
	Improved physical autonomy	• Independence • Empowerment • Self‐sufficiency • Increased confidence • Family support
Barriers	Device‐related barriers	• COVID apprehension • Uncomfortable nose piece • Feeling of nausea with mouthpiece • Exertional • Ringing in ears in initial sessions
	Issues with home‐based treatment	• Environmental distractions • Reduced commitment to exercise • Safety concerns • Technical issues • Self‐discipline • Lack of supervision • Limited feedback
	Lack of social communication	• Limited/no social interaction • Isolation
		• Lack of time/other responsibilities

**Figure 1:**
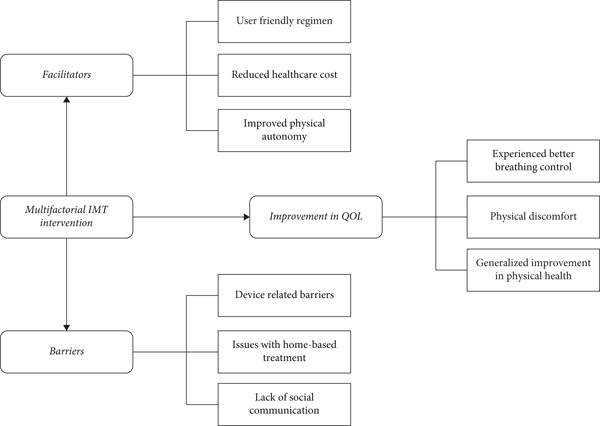
Theme chart showing themes and subthemes related to the patient′s perspective of using multifactorial IMT.

Overall, the patients were satisfied with this home‐based multifactorial intervention. However, they highlighted a few limitations, which can improve the level of satisfaction. Themes and subthemes are highlighted in Figure 1.

### 3.1. Theme 1: Improvement in HrQoL

All patients reported improvements in HrQoL after 12 weeks of multifactorial IMT intervention performed at home. The patients noted a substantial decrease in the level of dyspnea and reduced shortness of breath on activities that previously provoked the dyspnea.

One of the major benefits of these exercises is improvement in my shortness of breath. Previously, I experienced shortness of breath whenever I used to climb stairs and do speedy walk but recently, when my mother was admitted in hospital, I walked a lot more than usual but didn′t experience shortness of breath. (P6, M, 66 years)

They also reported that the comprehensive rehabilitation approach (i.e., IMT + OEP) improved their walking speed and reduced dyspnea and fatigue thereby improving functional independence. This not only improved their physical function but also helped in the improvement of mental well‐being through relaxation attainment and improved quality of sleep.

I feel better and more confident as this combination of exercises has not only reduced shortness of breath but also improved my physical health because I no more experience day to day discomfort and sleep better now. (P1, M, 56 years)

I used to feel exhausted all the time but now I feel myself as more energetic, independent, and more confident knowing how to control of my breath. (P8, M, 56 years)

There was also a generalized improvement in the physical health and well‐being of patients. The patients reported decreased muscle tension and stiffness which previously used to make them struggle all day thereby relieving the muscles aches.

The muscle tightness that previously used to stay with me all the time and drain me has now run away. These exercises have given me a major relief in terms of improved mobility. (P10, M, 63 years)

The day when I exercise is spent very well, but when I didn′t do exercise, the whole day spent too lazy, and I didn′t feel mentally relaxed. (P2, F, 56 years)

### 3.2. Theme 2: Facilitators to Multifactorial IMT

The patients were satisfied with the home‐based intervention as it was a combination of a user‐friendly and easy‐to‐use device‐based IMT training accompanied by a set of easy‐to‐perform OEP for balance and strength training.

The exercises are easy to perform at home where the environment is comfortable for me as I no longer must wait in the department for my turn as I used to do earlier. This saves time and unnecessary fatigue and exertion. (P9, M, 55 years)

The training diaries were maintained and weekly monitored to check adherence to the protocol, and the participants having adherence rate < 80*%* were considered as dropout from study.

I have weekly supervised session with my therapist which gives me confident that I am on track and keeps me motivated for the session throughout the week. (P11, F, 50 years)

The training was not only easy to perform and requiring minimal supervision but also reduced the healthcare costs associated with the supervised training sessions. It indirectly reduced the traveling and session costs thereby reducing the financial burden on the patients.

I no longer need to schedule my exercise sessions as per my son′s schedule of night shifts. (P5, F, 53 years)

Another facilitator of this intervention was the reduced healthcare cost associated with less frequent hospital visits saving their travel cost as well as cost of treatment sessions.

I experienced a significant relief in terms of cost after starting this home‐based treatment. (P3, M, 65 years)

Multifactorial IMT also improved the physical autonomy of the patients. The empowerment and independence the patient perceived, attained with the intervention, reduced the dependency on caregivers and made them accountable and self‐sufficient.

I feel more empowered as I can progress at my own pace. Now, I can try more challenging exercises and move forward when me and my therapist feel ready for it. (P2, F, 56 years)

### 3.3. Theme 3: Barriers to Multifactorial IMT

Although the adherence to the intervention was good and patients felt improvement in symptoms as well as quality of life with this home‐based intervention, still there were a few barriers highlighted by the patients. The most important being the initial difficulty in usage with device‐based treatment because of the uncomfortable nose clip and feeling of nausea and ringing of ears. This difficulty got settled as the patients became accustomed to this training.

Initially, I felt a constant beep in my ears and head and then felt dizziness but with time it got easier. (P7, M, 60 years)

When I started doing IMT, it felt like there was something in my ear and at times I felt nauseous also, but later, this feeling reduced and I got able to complete the required number of breaths easily. (P12, F, 55 years)

There was apprehension of patients related to COVID also which settled down with time.

Yes, I have to acknowledge my initial fears and concerns regarding COVID 19, when I understand the importance and need of treatment but the fear of contracting COVID with this breathing device was constantly in my mind. (P3, M, 65 years)

Patients reported it to be somewhat difficult to go for home‐based treatment as there is distraction in the home environment and it needs to be highly self‐directed, motivated, and self‐disciplined to continue with the treatment at home which becomes quite difficult at times.

Before starting treatment, I was quite happy with the idea of home‐based treatment performing exercises in the comfort of my own home at my own schedule but with time I realized the challenges of home‐based sessions, I had to find ways to overcome distractions and staying motivated. (P12, F, 55 years)

A few patients need monitoring and guidance to deal with safety and technical issues. Another challenge reported by the patients is the limited feedback on their performance as the patients underwent only one supervised session per week.

Although I have been guided properly, still I am a bit uncertain regarding what I am performing and miss the immediate supervision by my therapist, whose motivation kept my moral high. (P4, F, 52 years)

The patients also used to miss the social interaction with other patients at the hospital set‐up.

Though, I am satisfied with this home‐based approach, however, sometimes I find myself missing the social interaction with the fellow patients who were going through similar experiences. (P6, M, 66 years)

Another challenge of this intervention as reported by patients was the lack of time and other responsibilities also made them forget the sessions.

It was quite demanding at times, and I faced this challenge of balancing multiple responsibilities and managing time for the exercise session. I do recognize the importance of sessions but finding time for them was quite tough. (P11, F, 50 years)

## 4. Discussion

The study explored the perspective of patients with DPN regarding 12 weeks of multifactorial IMT intervention. The major themes identified were improvement in the quality of life, facilitators, and barriers to the home‐based multifactorial IMT, highlighting not only the positive impact of the intervention but also the challenges faced during the intervention.

Our results show patients′ perceived quality of life improved physically, mentally, and emotionally via the reduction in dyspnea levels, improved functional independence, better sleep, and decreased fatigue. These improvements are consistent with the improvements observed with IMT in patients with COVID‐19 [38] and advanced lung disease [39].

A thematic synthesis highlighting the nine domains of quality of life important to older adults, including autonomy, role and activity, health perception, relationships, attitude and adaptation, emotional comfort, spirituality, home and neighborhood, and financial security [40]. Out of these, almost all domains were improved after intervention in this qualitative study except for relationships and home and neighborhood. While the primary aim of this study was to explore patients′ perspectives, it is important to note that IMT improves the strength of the diaphragm and reduces metaboreflex activation and respiratory fatigue. These physiological benefits may contribute to the perceived improvements in daily functioning and overall HrQoL reported by the participants.

A higher level of self‐efficacy was reported by diabetes patients in the current study, as reported for COPD patients who reported higher self‐efficacy with IMT as compared to pulmonary rehabilitation [41].

Overall, the diabetic patients had a positive perception and experience of this intervention, like the patients who underwent the mobile health intervention [42]. However, the mobile health intervention patients faced the financial burden as a barrier compared to patients who underwent multifactorial IMT where financial cost was reduced [42]. The positive impact of this multifactorial intervention is not only attributed to IMT but also the OEP. This exercise program, based on easy‐to‐perform exercises, from a physical therapist′s perspective, improved physical functioning, promoted relaxation, improved self‐efficacy, and increased confidence [43].

As reported, multifactorial intervention is perceived to be a feasible and effective strategy to improve the quality of life of diabetes patients. The patients perceived it to be a user‐friendly regimen that is safe and easy to perform, requires minimal supervision, and can be easily incorporated into the patient′s routine. This is similar to those of a scoping review done on the use of nonmedical devices for chronic breathlessness that showed similar patient‐perceived facilitators like ease of use and comfort using the devices [44]. This finding also supports the usage of this intervention because in previous studies, lack of space/room for exercise was considered a barrier to PA [45].

The patients also reported it as a budget‐friendly intervention as it can save the travel and per‐session costs. As per the patients′ perception, the multifactorial IMT intervention also improved the physical autonomy of the patients, making them more empowered, independent, self‐sufficient, and confident. The patients recovering from COVID‐19 also regained their confidence and positivity and felt improvement physically and emotionally as well as cognitively [38].

Home‐based multifactorial IMT was found to be acceptable for diabetes patients except for the few challenges related to home‐based treatment and device‐related barriers. Despite the ease of technique, the patients reported initial difficulty in performing IMT, which was resolved later. The patients in the current study reported ringing of ears, dizziness, uncomfortable nose clips, and nauseous feeling when they started doing IMT; however, in another study, the patients reported feeling thirsty while performing it on initial days [41]. Neck pain in patients with arthritis and recurrent infections was also reported as a physical limitation to performing IMT [41]. The patients, when asked about how to improve the quality of intervention, expressed that nothing needed to be done as these difficulties were faced only initially, and it got better with time and practice.

In conclusion, this study is among the first studies to look at multifactorial IMT from a patient′s perspective and has proven the substantial effect of the intervention on the lives of the patients with diabetes. Being home‐based, the patients perceived the intervention as a convenient and accessible solution, particularly with transport/traveling issues. The patients consider it important as it can reduce healthcare costs and, at the same time, enhance their quality of life and contribute to better community health outcomes. The importance of this patient‐centered approach lies in its contribution to improved health outcomes, quality of life, healthcare cost reduction, better community health, and long‐term well‐being of patients [46].

Future studies need to investigate the perspectives of physical therapists and healthcare providers for improving adherence and overcoming the challenges as described by these patients.

## 5. Conclusion

The study revealed consistent themes regarding the patient′s perspective of home‐based multifactorial IMT, such as improvement in HrQoL, facilitators, and barriers to the treatment. The study highlights the effect of intervention from the patients′ view, leading to patient‐centered care and improved healthcare practices that ultimately contribute to the quality of care provided to the patients.

The patients experienced a profound improvement in their quality of life following intervention and reported noticeable perceived improvement in their general physical health. Improved physical autonomy, user‐friendly intervention requiring only minimal supervision, reduced healthcare costs, and easy incorporation in patients′ routines were the perceived facilitators, whereas device‐related barriers, distraction within the home environment, self‐regulation and discipline, and no social interaction were the perceived barriers identified that should be worked upon to facilitate incorporation of this multifactorial intervention in patients′ routines. Patients find multifactorial IMT to be an effective and user‐friendly approach to aid in the rehabilitation of patients with diabetes. While the findings provided detailed insights into patients′ perspectives, the study has a limitation of lacking a detailed record of the life activities of the patients during the intervention, especially their activity level and level of life stressors during the intervention. The study findings can be explored further in the future from the therapist/caregiver point of view to refine and improve exercise recommendations for patients with diabetes.

## 6. Limitations

The results of the study should be interpreted in the context of a few limitations. A potential limitation of this study is that the interviews were conducted by the same researcher who administered the intervention. Though efforts were made to minimize researcher‐induced bias, the dual role of the researcher could introduce a degree of subjectivity in the data. The individual interviews reflect individual experiences, thoughts, and perceptions of patients, but they may undermine the group interaction and the chances of gaining insight into the cause of individual differences that could have been attained with a focus group.

## Ethics Statement

Ethical approval has been taken from the ethics committee of Riphah International University, Islamabad, Pakistan (Ref # Riphah/RCRS/REC/13564). Written informed consent was taken from all the patients in the local language prior to the conduction of interviews.

## Disclosure

All authors have read and agreed to the submitted version of the manuscript.

## Conflicts of Interest

The authors declare no conflicts of interest.

## Author Contributions

Conceptualization, A.N.M. and S.S. Methodology, F.A.S. and S.S. Data collection and analysis, S.S., A.N.M., F.V.F., and F.A.S. Writing—original draft preparation, S.S. and F.V.F. Writing—review and editing, A.N.M., F.A.S., and F.V.F. Visualization, F.V.F. and S.S. Supervision, F.V.F. and A.N.M. Project administration, A.N.M. and S.S.

## Funding

No funding was received for this manuscript.

## Supporting information


**Supporting Information** Additional supporting information can be found online in the Supporting Information section. The supporting information provides a detailed checklist following the consolidated criteria for reporting qualitative research (COREQ) that ensures the study′s reporting is thorough, transparent, and aligned with recognized qualitative research standards [28].

## Data Availability

The datasets used and analyzed during the current study are available from the corresponding author on reasonable request.
